# Supplementary material for: Genome-Wide Analysis Reveals that PhoP Regulates Pathogenicity in Riemerella anatipestifer
Source: Microbiol Spectr. 2022 Oct 5;10(5):e01883-22. doi: 10.1128/spectrum.01883-22 (PMC9603813; doi:10.1128/spectrum.01883-22)
Supplement: Supplemental file 1 — Fig. S1 to S6 and Tables S1 to S4. Download spectrum.01883-22-s0001.pdf, PDF file, 1.2 MB [file spectrum.01883-22-s0001.pdf]

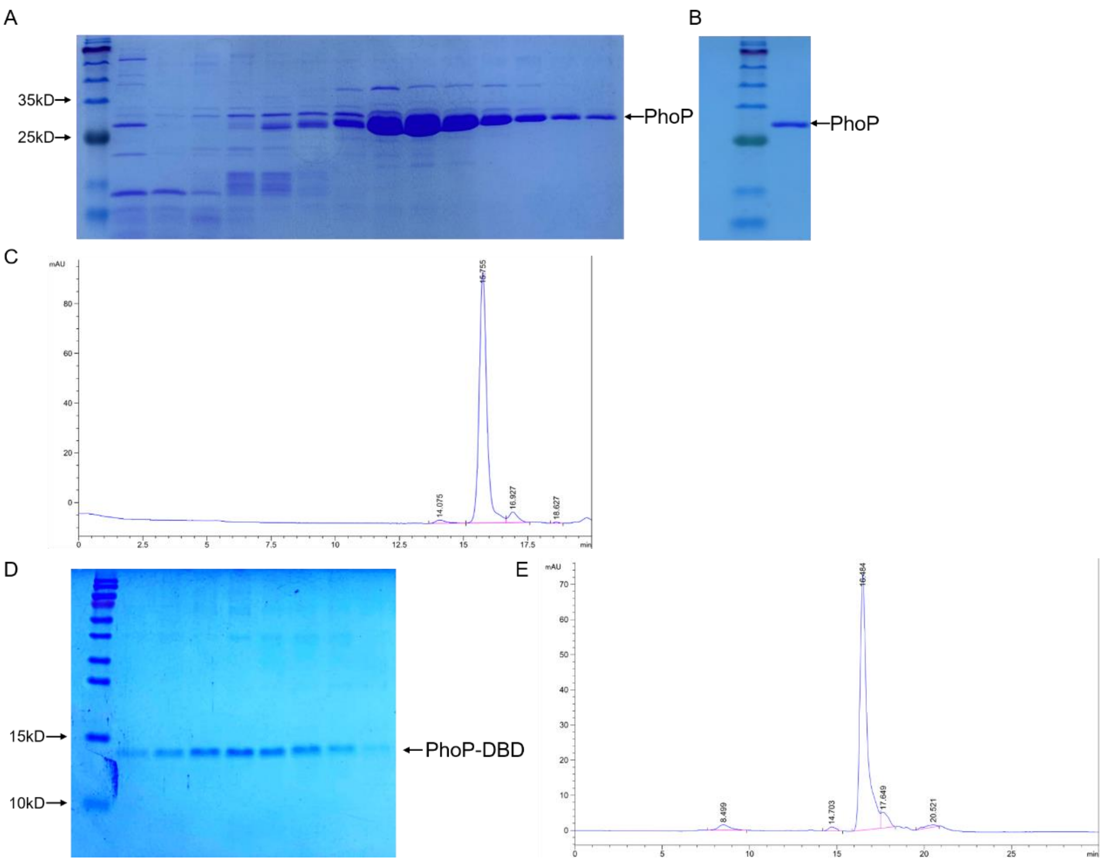

Figure S1. Expression of His$_6$-PhoP and His$_6$-PhoP-DBD. (A) Purification of His$_6$-PhoP by Ni-NTA affinity, and eluents with different concentrations of imidazole visualized using SDS-PAGE. His$_6$-PhoP is supposed to be 31.23 kD. (B) His$_6$-PhoP after ultrafiltration concentration using an ultrafilter (Merck KGaA, Germany). (C) Chromatography of His$_6$-PhoP by gel-filtration. The number above the peaks is the retention time. The purity of His$_6$-PhoP is 94.4232%. (D) Purification of His$_6$-PhoP-DBD by Ni-NTA affinity, and eluents with different concentrations of imidazole visualized using SDS-PAGE. His$_6$-PhoP is supposed to be 15.44kD. (E) Chromatography of His$_6$-PhoP-DBD by gel-filtration. The number above the peaks is the retention time. The purity of His$_6$-PhoP-DBD is 91.4392%.

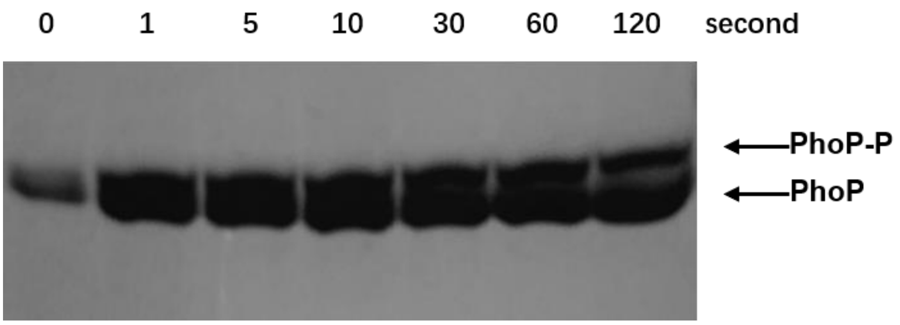

Figure S2. Identification of phosphorylation of PhoP *in vitro*. The phosphorylation of PhoP *in vitro* via a different duration of incubation with acetyl-phosphate was shown via Phos-tag SDS-PAGE followed by CBB staining.

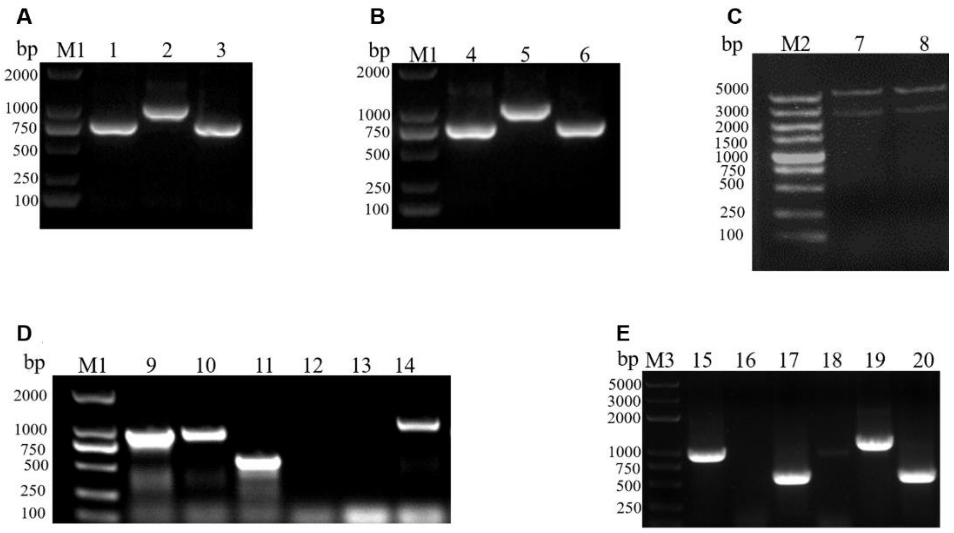

Figure S3. Construction of Δ*phoP* and Δ*phoR* mutant strains. (A) Homologous arm amplification for the *phoP* gene deletion. Lane 1: the left arm of *phoP*; Lane 2: spectinomycin resistance cassette; Lane 3: the right arm of *phoP*. (B) Homologous arm amplification for *phoR* gene deletion. Lane 4: the left arm of *phoR*; Lane 5: spectinomycin resistance cassette; Lane 6: the right arm of *phoR*. (C) Identification of the recombinant suicide plasmids for *phoP* and *phoR* gene deletion. Lane 7: *Kpn* I and *Sac* I digestion identification of the recombinant suicide plasmid for *phoP* gene deletion; Lane 8: *Kpn* I and *Sac* I digestion identification of recombinant suicide plasmid for *phoR* gene deletion. (D) *phoP* gene deletion strain identification by PCR amplification. 9. PCR product of *phoR* gene in RA-YM; 10. PCR product of *phoR* gene in Δ*phoP*; 11. PCR product of *phoP* gene in RA-YM; 12. PCR product of *phoP* in Δ*phoP*; 13. PCR product of Spec cassette in RA-YM; 14. PCR product of Spec cassette in Δ*phoP*. (E) Identification of *phoR* gene deleted strain by PCR amplification. 15. PCR product of *phoR* gene in RA-YM; 16. PCR product of Spec cassette in RA-YM; 17. PCR product of *phoP* gene in RA-YM; 18. PCR product of *phoR* in Δ*phoR*; 19. PCR product of Spec cassette in Δ*phoR*; 20. PCR product of

phoP gene in Δ*phoR*; M1: DL2000 DNA Marker; M2 and M3: DL5000 DNA Marker.

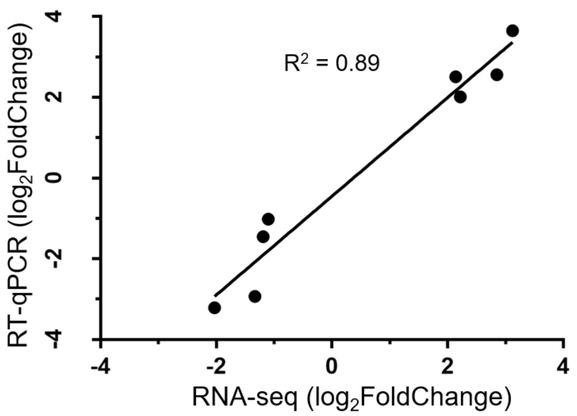

Figure S4. Validation of RNA-seq data for the representative candidate target genes by RT-qPCR. Correlation of gene expression between RT-qPCR and RNA-seq data. 8 genes from Table 1 and 2 are selected, and primers are listed in S2 Table.

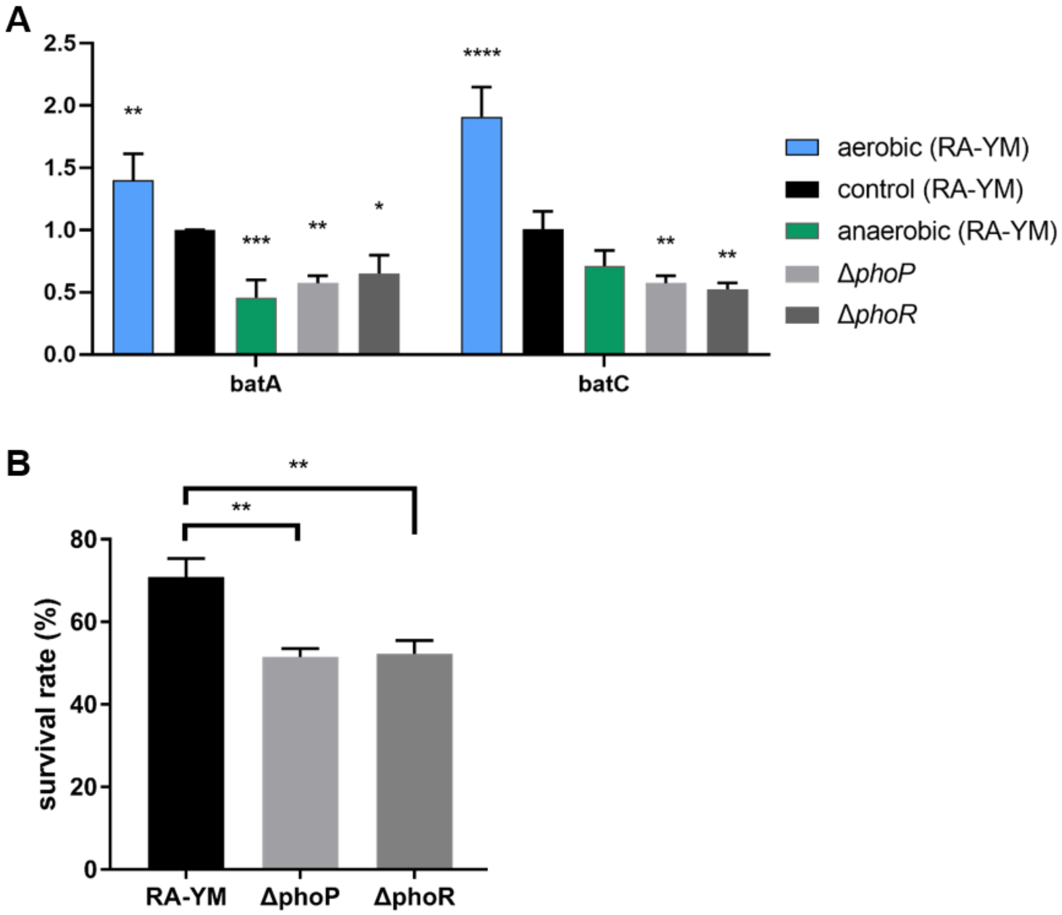

Figure S5. mRNA levels of *batA* and *batC* in different strains or different oxygen content treatments and survival rate after treatment with hydrogen peroxide. RA-YM and derivates were grown in TSB to exponential phase, and the bacterial were harvested and resuspended in new TSB with OD_{600} adjusted to 1.0. (A) The following conditions were applied for RA-YM: aerobic (10 mM H_2O_2 was added to 1 mL RA-YM), control (RA-YM), anaerobic (culture 1 mL RA-YM in anaerobic incubator). All adjusted bacteria were grown for 2h and harvested for RNA extraction. *recA* was chose as the reference gene. (B) The survival rate of RA-YM and derivates after treatment with 10 mM H_2O_2, performed from three independent experiments. p (*) < 0.0332, p (**) < 0.0021, p (***) < 0.0002, p (****) < 0.0001.

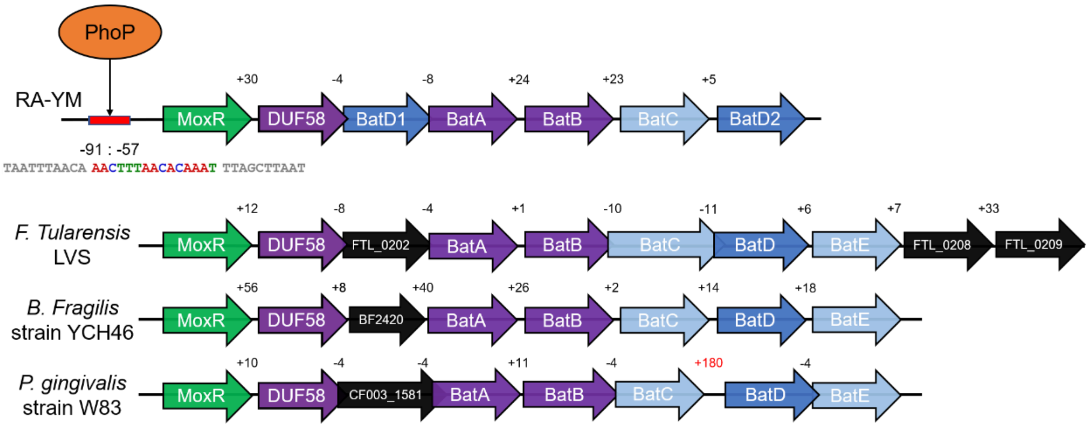

Figure S6. Schematic organization of Bat operon. The upper line showed that PhoP directly binds to the upstream region of *moxR* to enhance transcription of Bat operon, and the PhoP-binding site showed in red with the binding sequence shown below. Bat operon of *F. Tularensis* LVS, *B. Fragilis* strain YCH46 and *P. gingivalis* strain W83 were shown for comparison. Distance between adjacent genes is shown above the sequence (negative number indicates the number of overlapping bases, positive number indicates the number of separated bases).

S1 Table. Strains and plasmids used in this study.

*S*trains	Description	Source/reference
E. coli DH5α	Plasmid propagation strain	Weidi Biotech Co., Ltd.
E. coli BL21 (DE3)	Protein overexpression strain	Weidi Biotech Co., Ltd.
E. coli χ7213	Diaminopimelic acid autotrophic strain used in transconjugation. *λpir*	Laboratory collection
E. coli DH5α *λpir*	Plasmid propagation strain. *λpir*	Weidi Biotech Co., Ltd.
RA-YM	*Riemerella anatipestifer* wild type strain serotype 1, strong virulence	Laboratory collection
Δ*phoP*/*phoR*	*phoP*/*phoR* operon deletion strain, $Spec^R$	(1)
Δ*phoP*	*phoP* gene deletion strain, $Spec^R$	This study
Δ*phoR*	*phoR* gene deletion strain, $Spec^R$	This study
**Plasmids**	**Description**	**Source/reference**
pET-28a	Protein overexpression strain	Laboratory collection
pET-28a-PhoP	the plasmid used to express recombinant His_6-PhoP	This study
pET-28a-PhoP-DBD	the plasmid used to express recombinant His_6-PhoP-DBD	This study
pRE112	Suicide vector, *oriT*, *oriV*, *sacB*, Cm^R	Laboratory collection
pRE-PhoP-LSR	pRE112 containing the fusion fragment consisting of left and right arm of *phoP* and spectinomycin resistance gene	This study
pRE-PhoR-LSR	pRE112 containing the fusion fragment consisting of left and right arm of *phoR* and spectinomycin resistance gene	This study
pIC 333	source of spectinomycin resistance cassette	Laboratory collection

Reference

1. Wang Y, Lu T, Yin X, Zhou Z, Li S, Liu M, Hu S, Bi D, Li Z. 2017. A Novel RAYM_RS09735/RAYM_RS09740 Two-Component Signaling System Regulates Gene Expression and Virulence in Riemerella anatipestifer. Front Microbiol 8:688.

S2 Table. Oligonucleotides used in this study.

Primers	Primer sequence (5'-3')	Purpose
**Primers for constructing suicide plasmids and *phoP* gene mutant**		
phoP L-F	CT**GGTACC**CACTATTGCTGATGAGGTTTACCTTGAAAATCAATCTAAAG	amplification of left arm of *phoP*
phoP L-R	*AACGTGAGTTTTCGTTCCACTG* CTTTGGTCATCTTCTACTAATAATATCCTGTTGCTCAT	
phoP Spec F	ATGAGCAACAGGATATTATTAGTAGAAGATGACCAAAG*CAGTGGAACGAAAACTCACGTT*	amplification of spectinomycin resistance cassette for constrution of *phoP*- LSR fragment
phoP Spec R	TTAATTTTTAACTAGAAGCCTAAACCCTTCCCCG*CAGTAGTTTTAAAAGTAAGCACCTG*	
phoP R-F	*CAGGTGCTTACTTTTAAAACTACTGTT* CGGGGAAGGGTTTAGGCTTCTAGTTAAAAATTAA	amplification of right arm of *phoP*
phoP R-R	A**GAGCTC**GCACCACTCATTATGATTTTCTTTTGTATTTATTGTTAGAG	
**Primers for constructing suicide plasmids for *phoP* and *phoR* gene mutant**		
phoR L-F	CT**GGTACC**GCCTTGGTTTCTTACTCTTTCATCCATCATAGAG	amplification of left arm of *phoR*
phoR L-R	*AACGTGAGTTTTCGTTCCACTG* ATAGATATAATAGGAATAAATTTGTTACGCAC	
phoR Spec F	GTGCGTAACAAATTTATTCCTATTATATCTAT*CAGTGGAACGAAAACTCACGTT*	amplification of spectinomycin resistance cassette for construction of *phoR*- LSR fragment
phoR Spec R	TTAAATCATTGGAAGTTTTACAATAAAGGTAC*CAGTAGTTTTAAAAGTAAGCACCTG*	
phoR R-F	*CAGGTGCTTACTTTTAAAACTACTGTT* GTACCTTTATTGTAAAACTTCCAATGATTTAA	amplification of right arm of *phoR*
phoR R-R	A**GAGCTC**ACTTTATCTATCATCTGTAGAACTCTATTTGC	
**Primers for identifying the *phoP* and *phoR* genes mutant**		
phoP ID F	ATGAGCAACAGGATATTATTAGTAGAAGATGACCAAAG	detection of deletion of *phoP*
phoP ID R	ATTTTTAACTAGAAGCCTAAACCCTTCCCCGTGTACATT	
phoR ID F	AGGAATATTATAGCTCTATTGAAGAAGAATTCGC	detection of deletion of *phoR*
phoR ID R	CATTTCGTTCCTCTCTAACTTTGACATATTG	
**Primers for constrution the overexpression of PhoP and PhoP-DBD**		
PhoP-F	CCG**GAATTC**AGCAACAGGATATTATTAGTAGAAGATGACC	amplification of PhoP fragment
PhoP-R	CCG**GGATCC**TTAATTTTTAACTAGAAGCCTAAACCCTTCCCC	
PhoP-DBD-F	CCG**GAATTC**GAGCAATTTAGCATTAGCAATATAGAG	amplification of PhoP-DBD fragment
PhoP-DBD-R	CCG**GGATCC**TTAATTTTTAACTAGAAGCCTAAACCC	
**Primers for EMSA probes**		
biotin-phoPR motif 1	AAACTCATAATAATCTGTTAAAAAACTCGTTTTTA	construction of the probe for PhoPR
phoPR motif 2	TAAAAACGAGTTTTTTAACAGATTATTATGAGTTT	
biotin-moxR motif 1	TAATTTAACAAACTTTAACACAAATTTAGCTTAAT	construction of the probe for MoxR
moxR motif 2	ATTAAGCTAAATTTGTGTTAAAGTTTGTTAAATTA	
biotin-KYF39_06865 motif 1	CGTTAAAAAATAAAGCGAGGTTTTGTTGTTTTAAA	construction of the probe for KYF39_06865
KYF39_06865 motif 2	TTTAAAACAACAAAACCTCGCTTTATTTTTTAACG	
biotin-dnaG motif 1	GTTATCAAATTTTTATGATAAAATTAGATGCTGTT	construction of the probe for DnaG
dnaG motif 2	AACAGCATCTAATTTTATCATAAAAATTTGATAAC	
biotin-dedA motif 1	ATCTATAAAGAGATTTAACAAATTTTATAATTCTA	construction of the probe for DedA
dedA motif 2	TAGAATTATAAAATTTGTTAAATCTCTTTATAGAT	
biotin-transposase 1	TGAAAATTTCAAATTTAACTAAAGCTTATAGCAAT	construction of the probe for KYF39_00915
transpoase 2		
biotin-transporter 1	CAAAAAATATTTCATTAACAGAATAGACAATCATA	construction of the probe for KYF39_00905
transporter 2		
biotin-OMPβB 1	ATAAAGTGGCAAATTTAACTTTAATAATTAAAAGA	construction of the probe for KYF39_07405
OMPβB 2		
**Primers for rt-qPCR**		
recA qPCR F	TTTAGGCGTAAGTGCTGCCA	qPCR for *recA*
recA qPCR R	CCAGATAATGGAGAGCAGGCA	
phoP	ACGGCGAGGAAGGTCTTAAA	qPCR for *phoP*
phoP-2	CGTCTGCACCTAACTGATAACC	
phoR	GCCCCAAACCTTGACCTTTT	qPCR for *phoR*
phoR-2	GGTATGGGAATGGAGCCTCA	
KYF39_09010	AGAGTGCCATTGACTTCGGA	qPCR for *batA*
KYF39_09010-2	ACAGCTACCGCCAAACCATC	
KYF39_09020	ACAGGACAGACGCCTAAACC	qPCR for *batC*
KYF39_09020-2	GCTCTTTGCTTTCCAAGCGT	
KYF39_00915	AGTTTTGTTTACCCAGGCGTTG	qPCR for *KYF39_00915*
KYF39_00915-2	TGTCCTAAAGGAAAGGTCTGCTG	
KYF39_00905	AGCTTTGTAATGATTTGGCGGT	qPCR for *KYF39_00905*
KYF39_00905-2	ACGGCTCCACACTTTTCCTT	
KYF39_08995	GTGCCTGGCTTGGCAAAAA	qPCR for *moxR*
KYF39_08995-2	CTGCAGGTAGAAGGTCTGGC	
KYF39_07405	AGCTTATTCAGAAAGTAACTCGTCA	qPCR for OMP-βB
KYF39_07405-2	CGGCAACGGAAGTCCTACAT	
KYF39_08580	CTTGCTGTGCTCCTGTGGTA	qPCR for TBDR
KYF39_08580-2	CGGCTAACTAACGACACCGT	
KYF39_09485	GGCTTTAATCCCTGCAGCATC	qPCR for *dnaG*
KYF39_09485-2	CGAGTTCTGGAACGGCACTT	

Restriction sites are sited in bold, and overlap part for overlap PCR are sited in italic.

S3 Table. 59 DEGs both in Δ*phoP* and Δ*phoR*

Gene	log2FoldChange in ΔPhoP	log2FoldChange in ΔPhoR	Annotation	candidate target genes
KYF39_00135	1.17	1.79	hypothetical protein	
KYF39_00620	1.36	1.72	energy transducer TonB	
KYF39_00905	3.12	1.72	ABC transporter ATP-binding protein	**yes**
KYF39_01685	1.08	1.52	PorT family protein	
KYF39_02145	-1.19	-1.77	hypothetical protein	
KYF39_02185	2.32	2.05	cold-shock protein	
KYF39_02190	2.71	2.37	cold shock domain-containing protein	
KYF39_02995	-4.31	-3.35	septal ring lytic transglycosylase RlpA family protein	
KYF39_04280	-1.02	-1.61	hypothetical protein	
KYF39_05510	1.78	2.13	adenosine deaminase	**yes**
KYF39_05585	-1.02	-1.17	hypothetical protein	**yes**
KYF39_05595	-1.72	-1.19	efflux RND transporter permease subunit	
KYF39_05775	1.09	-10.19	HAMP domain-containing histidine kinase	**yes**
KYF39_06320	-1.25	-1.01	DinB family protein	
KYF39_06325	1.13	-1.11	hypothetical protein	
KYF39_06420	-3.41	-2.77	C40 family peptidase	
KYF39_06505	1.25	1.19	hypothetical protein	**yes**
KYF39_06925	1.38	1.34	DUF4407 domain-containing protein	**yes**
KYF39_07405	2.22	2.11	outer membrane beta-barrel protein	**yes**
KYF39_07620	-1.18	-1.12	tRNA-Gly	
KYF39_07675	1.15	1.25	thrombospondin type 3 repeat-containing protein	
KYF39_07940	1.15	2.09	zinc-dependent metalloprotease	
KYF39_08245	1.86	1.46	hypothetical protein	
KYF39_08555	1.04	1.27	FAD:protein FMN transferase	
KYF39_08580	2.85	4.47	TonB-dependent receptor	**yes**
KYF39_08585	1.10	1.03	alpha/beta hydrolase	**yes**
KYF39_08895	2.85	2.65	SusC/RagA family TonB-linked outer membrane protein	
KYF39_08900	3.16	2.96	RagB/SusD family nutrient uptake outer membrane protein	
KYF39_08905	3.09	2.73	putative zinc-binding metallopeptidase	
KYF39_08910	3.09	2.77	DUF4302 domain-containing protein	
KYF39_08915	3.12	2.56	hypothetical protein	**yes**
KYF39_08920	3.19	2.59	DUF4856 domain-containing protein	
KYF39_08995	-2.03	-2.05	AAA family ATPase (MoxR)	**yes**
KYF39_09000	-2.16	-2.38	DUF58 domain-containing protein	
KYF39_09005	-2.05	-2.40	BatD family protein	
KYF39_09010	-1.33	-2.03	VWA domain-containing protein (BatA)	**yes**
KYF39_09020	-1.44	-2.08	tetratricopeptide repeat protein (BatC)	**yes**
KYF39_09130	-1.81	-1.59	GLPGLI family protein	**yes**
KYF39_09165	-1.14	-1.60	GLPGLI family protein	
KYF39_09225	-1.37	-2.50	hypothetical protein	
KYF39_09235	-1.82	-3.29	hypothetical protein	
KYF39_09245	-1.14	-1.67	hypothetical protein	
KYF39_09250	-1.33	-3.04	hypothetical protein	
KYF39_09270	-1.83	-2.74	GLPGLI family protein	**yes**
KYF39_09285	-1.71	-3.69	SEL1-like repeat protein	
KYF39_09290	-1.92	-2.61	hypothetical protein	**yes**
KYF39_09295	-1.81	-3.94	hypothetical protein	
KYF39_09300	-1.18	-1.94	hypothetical protein	
KYF39_09305	-1.62	-2.80	hypothetical protein	
KYF39_09310	-1.22	-2.05	HXXEE domain-containing protein	**yes**
KYF39_09340	-1.68	-3.32	hypothetical protein	
KYF39_09385	-1.43	-2.05	nucleotidyltransferase domain-containing protein	
KYF39_09395	-1.20	-2.74	hypothetical protein	
KYF39_09405	-1.12	-1.62	hypothetical protein	**yes**
KYF39_09410	-1.72	-3.01	hypothetical protein	**yes**
KYF39_09425	-1.29	-2.92	hypothetical protein	**yes**
KYF39_09440	-1.13	-2.02	GLPGLI family protein	**yes**
KYF39_09485	-1.19	-1.29	DNA primase (DnaG)	**yes**
KYF39_09945	-1.27	-1.70	hypothetical protein	**yes**

S4 Table. Death of ducklings inoculated the Δ*phoP* , Δ*phoR* and RA-YM strain

**CFU**	**Death/Total**		
	RA-YM	Δ*phoP*	Δ*phoR*
1.0×10^5	8/10	0/10	0/10
1.0×10^6	9/10	0/10	0/10
1.0×10^7	10/10	0/10	0/10
1.0×10^8	10/10	1/10	0/10
1.0×10^9	10/10	3/10	1/10
Control		0/10	
